# Visual quality assessment of the liver graft by the transplanting surgeon predicts postreperfusion syndrome after liver transplantation: a retrospective cohort study

**DOI:** 10.1186/s12871-018-0493-9

**Published:** 2018-03-09

**Authors:** Felix Kork, Alexandra Rimek, Anne Andert, Niklas Jurek Becker, Christoph Heidenhain, Ulf P. Neumann, Daniela Kroy, Anna B. Roehl, Rolf Rossaint, Marc Hein

**Affiliations:** 1 Department of Anaesthesiology, Medical Faculty, RWTH Aachen University, Pauwelsstrasse 30, 52074 Aachen, Germany; 2 Department of General, Visceral and Transplantation Surgery, Medical Faculty, RWTH Aachen University, Pauwelsstrasse 30, 52074 Aachen, Germany; 3 Department of General, Visceral and Oncological Surgery, Sana Hospital Gerresheim, Gräulinger Strasse 120, 40625 Düsseldorf, Germany; 4 Department of Medicine III, Medical Faculty, RWTH Aachen University, Pauwelsstrasse 30, 52074 Aachen, Germany

**Keywords:** Steatosis, Hyponatremia, Cold ischemia time

## Abstract

**Background:**

The discrepancy between demand and supply for liver transplants (LT) has led to an increased transplantation of organs from extended criteria donors (ECD).

**Methods:**

In this single center retrospective analysis of 122 cadaveric LT recipients, we investigated predictors of postreperfusion syndrome (PRS) including transplant liver quality categorized by both histological assessment of steatosis and subjective visual assessment by the transplanting surgeon using multivariable regression analysis. Furthermore, we describe the relevance of PRS during the intraoperative and postoperative course of LT recipients.

**Results:**

53.3% (*n* = 65) of the patients suffered from PRS. Risk factors for PRS were visually assessed organ quality of the liver grafts (acceptable: OR 12.2 [95% CI 2.43–61.59], *P* = 0.002; poor: OR 13.4 [95% CI 1.48–121.1], *P* = 0.02) as well as intraoperative norepinephrine dosage before reperfusion (OR 2.2 [95% CI 1.26–3.86] per 0.1 μg kg^− 1^ min^− 1^, *P* = 0.01). In contrast, histological assessment of the graft was not associated with PRS. LT recipients suffering from PRS were hemodynamically more instable after reperfusion compared to recipients not suffering from PRS. They had lower mean arterial pressures until the end of surgery (*P* < 0.001), received more epinephrine and norepinephrine before reperfusion (*P* = 0.02 and *P* < 0.001, respectively) as well as higher rates of continuous infusion of norepinephrine (*P* < 0.001) and vasopressin (*P* = 0.02) after reperfusion. Postoperative peak AST was significantly higher (*P* = 0.001) in LT recipients with PRS. LT recipients with intraoperative PRS had more postoperative adverse cardiac events (*P* = 0.05) and suffered more often from postoperative delirium (*P* = 0.04).

**Conclusions:**

Patients receiving ECD liver grafts are especially prone to PRS. Anesthesiologists should keep these newly described risk factors in mind when preparing for reperfusion in patients receiving high-risk organs.

## Background

According to the Organ Procurement and Transplantation Network of the United States (US) Department of Health and Human Services, the numbers of liver transplantations (LT) in the US have constantly been rising since the beginning of LT [1]: In 2016, a total of 7841 LTs were performed in the US. By contrast, the numbers of LTs in Germany and in the Eurotransplant region have been decreasing since a peak in 2010 [2, 3]. The decreasing organ donations combined with the persisting high morbidity and mortality of patients on the waiting list has led to a discrepancy between organ supply and demand [2, 4] and therefore to the more frequent acceptance extended criteria donors (ECD) to the pool of eligible donors [5, 6].

The most critical intraoperative moment for the anesthesiologist during LT is the reperfusion of the liver graft. An immediate and severe complication following reperfusion is hemodynamic instability, the so called post-reperfusion syndrome (PRS). PRS is defined as a decrease of mean arterial pressure (MAP) of more than 30% during the first 5 min after reperfusion and continuing for at least 1 min [7–12]. PRS occurs in around 10–60% of LT recipients [8, 9, 13]. Several risk factors for PRS have been described: Older donor age, higher donor risk index, longer cold ischemic time (CIT), severity of the recipient’s liver disease, operation time and technique, hemodynamics at time of reperfusion, and steatosis of the graft organ [8, 13–20]. The mechanisms of PRS appear to be complex and not fully understood [10, 21, 22]. In addition, the transplantation of ECD livers has led to a decrease in mortality for LT recipients on the wait lists at the cost of increased perioperative complication [23]. Both these factors have made it difficult to predict PRS. In particular, the quality of the donor organ and its role as risk factor for the occurrence of PRS as well as its associations with patients’ outcome have been neglected in the past.

## Methods

### Aim, design and setting of the study

We therefore conducted a retrospective analysis of all LTs performed at our center. Primary aim of this study was to identify predictors of PRS including transplant liver quality assessed by both subjective visual assessment by the transplanting surgeon and histological assessment of steatosis. Secondary aim of this study was to describe the relevance of PRS during the intraoperative and postoperative course of LT recipients.

### Patients and management

All patients receiving cadaveric liver transplantation from the beginning of our center’s newly established liver transplantation program in May 2010 until January 1st, 2014 were considered eligible for inclusion. Patients were excluded in case histological donor data or intraoperative data were incomplete or in case of intraoperative severe adverse events prior to reperfusion. Liver transplantation was performed using an extracorporeal venovenous/portal venous bypass. Anesthesiological management, bypass and surgical procedures, as well as the immune suppression regimen have already been described by Moosdorf and colleagues [24]. Anesthesiologists did not follow a specific coagulation or transfusion management protocol: Patient received at maximum 1 Liter of balanced electrolyte solution and volume replacement was subsequently conducted with FFP in order to anticipate the coagulation disorder. Transfusion triggers for RBCs were tailored to the patient’s comorbidities and provided at the discretion of the providing anesthesiologist. Our department’s standard operating procedure (SOP) for LT includes a TEM after induction, 15–30 and 45–60 min after reperfusion, seeking to keep normal coagulation parameters [25].

### Data

#### Donor data

The following data were abstracted from the covering letter of the donor organ: donor age, donor body mass index, donor blood sodium concentration, donor alanine transaminase (ALT) blood concentration, donor aspartate transaminase (AST) blood concentration, donor bilirubin blood concentration, as well as warm ischemia time (WIT) and cold ischemia time (CIT).

#### Donor organ assessment

Donor livers were macroscopically assessed regarding their fat content by the implanting surgeon before the recipient operation on the preserved cold graft. Organs were categorized as either good, acceptable or poor, according to EuroTransplant criteria. In addition, histological assessment of the donor organ was conducted by the explanting center (or if missing by the in-house pathology department). Organs were categorized in three categories depending on macrovesicular fat content (≥ one intracellular vacuole displacing organelles): Grade 1 – fat content 0–29%, grade 2 – fat content 30–59%, grade 3 – fat content ≥60% [26, 27].

#### Recipient data

The following data were abstracted from the patient’s medical chart, as recorded at the time of evaluation for being listed for transplantation: Recipient age, recipient diagnosis leading to transplantation, portal hypertension (PoHT; defined by either esophageal varices, thrombopenia or splenomegaly), laboratory model of end stage liver disease score (labMELD; 10 x (0,957 x In(serum creatinine) + 0,37 x In(serum creatinine) + 1,12 x In (international normalized ratio[INR]) + 0,643)) [28], and the need for renal replacement therapy. From the electronic patient data management system, concentrations of serum bilirubin, serum AST, serum ALT, serum creatinine, international normalized ratio (INR) as well as renal replacement therapy were abstracted after admission closest to the beginning of surgery (preoperatively), at ICU admission immediately after surgery (postoperatively) and on postoperative day (POD) 1,3,7 and 14.

The following data were abstracted from the paper-based anesthesia protocol: Heart rate, mean blood pressure (arterial line), boli of norepinephrine and epinephrine at time of reperfusion, infusion rate of norepinephrine, infusion rate of epinephrine, infusion rate of vasopressin. These values were abstracted at induction of anesthesia, skin incision, beginning of the anhepatic phase, and at 5, 10, 15, 30 and 60 min after reperfusion as well as at the end of the surgery. Furthermore, the number of intraoperatively administered red blood cell concentrates (RBCs), platelet concentrates (PCs), fresh frozen plasmas (FFPs), the amount of intraoperatively administered fibrinogen, prothrombin complex concentrate (PCC), the occurrence of hyperfibrinolysis (by thrombelastometry [TEM]), asystole and cerebral edema (temporary mydriasis after reperfusion) were extracted from the anesthesia protocol.

From the patients chart we abstracted the following data from the postoperative period: primary nonfunction (PND; re-transplantation or death within 7 days), early allograft dysfunction (bilirubin ≥10 mg/dl on post-operative day (POD) 7 and/or INR ≥1.6 on POD 7 and/or AST or ALT > 2000 IU/L within the first 7 days), acute rejection (clinical diagnosis), surgical revisions, retransplantation, sepsis, need for renal replacement therapy (RRT), adverse cardiovascular events (asystole, resuscitation, non ST elevation myocardial infarction, heart failure), adverse central nervous events (delirium, intracranial hemorrhage, seizures), duration of mechanical ventilation, intensive care unit (ICU) length of stay (LOS), hospital LOS, and death.

#### Postreperfusion syndrome

PRS was defined as fulfillment of at least one of the following criteria: (1) Decrease in mean arterial pressure (MAP) of at least 30% at time of reperfusion, (2) administration of an intravenous bolus of norepinephrine > 2 μg kg body weight (BW) ^-1^, (3) increase of continuous norepinephrine infusion of ≥0.1 μg kg BW^− 1^ within 5 to 30 min after reperfusion, or (4) initiation of continuous vasopressin infusion after reperfusion. According to our department’s SOP, PRS was treated as follows: (i) 0.5 mg atropine before reperfusion if heart rate < 80, (ii) NE boli and NE infusion to maintain MAP, (iii) epinephrine boli and infusion in case of significant bradycardia with hypotension and decrease of SVO_2_ during reperfusion, (iv) infusion of vasopressin if high doses of NA are necessary or NA therapy ineffective.

### Statistics

Categorical data with two categories each were tested using Fisher’s exact test, with more than three categories with the Chi squared test with Yates correction. Since the sample size was > 100, central limit theorem applies and normal distribution of continuous variables was assumed without testing. Groupwise comparison of continuous variables were therefore conducted using the *t*-test for independent samples. Changes of continuous variables over time were compared using a repeated measures ANOVA test. If a time/variable-interaction was detected by the ANOVA, a post-hoc *t*-test was conducted for each time point. Survival analysis were conducted plotting Kaplan-Meier curves and these were compared using the Log Rank test. Agreement between visual organ assessment by the surgeon and histopathological steatosis grading was quantified by calculating an unweighted Cohen’s kappa. Binary logistic regression analysis was used to determine independent factors predicting postreperfusion syndrome. Variables reaching a level of significance (*P* ≤ 0.05) when univariably tested, were introduced into the multivariable logistic regression model. Statistical analyses were conducted using IBM SPSS 22, figures were created using GraphPad Prism 6.0. A two-sided *p*-value ≤0.05 was considered statistically significant.

## Results

### Patients

**Fig. 1:**
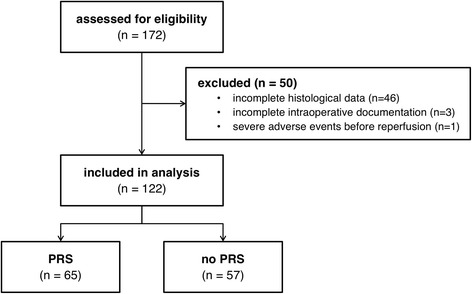
Patients. Flow chart of patient inclusion. PRS: postreperfusion syndrome

A total of 172 patients received a liver transplantation during the study period. 50 patients were excluded due to incomplete histological data (*n* = 46), incomplete intraoperative documentation (*n* = 3) or due to severe adverse events prior to reperfusion (*n* = 1; need for cardiopulmonary bypass due to laceration of the inferior caval vein). A total of 122 patients were analyzed (Fig. 1).

Clinical characteristics of the study population are depicted in Table 1. Recipients of LT had a mean age of 55 (54.9 ± 9.8) and a mean labMELD of 20 (19.8 ± 10.3). The most frequent reason for transplantation was cirrhosis (57 of 122; 46.7%) followed by tumor (25 of 122; 20.5%). Organ donors had a mean age of 55 (55.2 ± 16.2), were 46.7% female, had a mean BMI of 29 (28.8 ± 7.2), and serum sodium, ALT, AST, bilirubin within the reference range. The mean CIT of the donor organs was 8 h (8.3 ± 2.3), mean WIT was 44 min (43.7 ± 7.5).

**Table 1:** Clinical characteristics. of liver organ donors (top) and liver transplant recipients (bottom) of 122 single center liver transplantations (LTs)

	All LTs (*n* = 122)	PRS (*n* = 65)	no PRS (*n* = 57)	*P*
Donor characteristics				
Age [years]	55.2 ± 16.2	57.1 ± 15.1	53.1 ± 17.2	0.18
Sex [female]	57 (46.7%)	32 (49.2%)	24 (42.1%)	0.42
BMI [kg/cm^2^]	28.8 ± 7.2	31.1 ± 8.2	26.4 ± 4.8	< 0.001
Sodium [mmol/L]	147.7 ± 8.2	149.1 ± 7.8	146.1 ± 8.4	0.04
ALT [U/L]	114.2 ± 289.6	106.7 ± 223.2	122.8 ± 352.4	0.77
AST [U/L]	124.4 ± 237.8	115.9 ± 182.1	134.1 ± 290.0	0.69
Bilirubine [mg/dL]	0.86 ± 1.23	0.80 ± 1.33	0.93 ± 1.13	0.59
Cold ischemic time [h]	8.3 ± 2.3	8.7 ± 2.3	7.8 ± 2.3	0.04
≥ 10 h [n]	23 (18.9%)	17 (26.2%)	6 (10.5%)	0.02
Warm ischemic time [min]	43.7 ± 7.5	44.9 ± 6.7	42.3 ± 8.2	0.06
Recipient characteristics				
Age [years]	54.9 ± 9.8	55.2 ± 9.8	54.5 ± 9.8	0.69
Sex [female]	43 (35.2%)	21 (32.3%)	22 (38.6%)	0.57
BMI [kg/cm^2^]	27.6 ± 5.3	27.3 ± 5.5	28.1 ± 5.0	0.41
Diagnosis [n]				
acute liver failure	12 (9.8%)	10 (15.4%)	2 (3.5%)	0.03
acute on chronic	5 (4.1%)	1 (1.5%)	4 (7.0%)	0.18
cirrhosis	57 (46.7%)	20 (30.8%)	37 (64.9%)	< 0.001
tumor	25 (20.5%)	14 (21.5%)	11 (19.3%)	0.82
graft failure	7 (5.7%)	2 (3.1%)	5 (8.8%)	0.25
others (PSC, polycystic)	16 (13.1%)	10 (15.4%)	6 (10.5%)	0.59
portal hypertension	85 (69.7%)	52 (80.0%)	33 (57.9%)	0.01
labMELD	19.8 ± 10.3	20.0 ± 9.9	19.6 ± 10.8	0.82
Creatinine preop [mg/dl]	1.71 ± 1.49	1.75 ± 0.23	1.66 ± 0.15	0.14
preoperative RRT [n]	15 (12.3%)	8 (12.3%)	7 (12.3%)	1.00

*LT* Liver transplantation, *PRS* postreperfusion syndrome, *BMI* body mass index, *ALT* Alanine transferase, *AST* Aspartat transferase, *labMELD* laboratory Model of end-stage liver disease score, *RRT* renal replacement therapy

Clinical characteristics of liver organ donors (top) and liver transplant recipients (bottom) of 122 single center LTs. The characteristics are displayed for the whole study population (left) and by occurrence of postoperative reperfusion syndrome; mean and standard deviation (SD) or frequencies

### Postreperfusion syndrome

Of the 122 liver transplant recipients, 65 (53.3%) developed PRS (Table 1). Patients developing PRS received an organ more often due to acute liver failure (10 of 65 vs. 2 of 57, 15.4% vs. 3.5%, *P* = 0.03) and less often due to cirrhosis (20 of 65 vs. 37 of 57, 30.8% vs. 64.9%, *P* < 0.001) compared to recipients not developing PRS and suffered more often from PoHT (52 of 65 vs. 33 of 57, 80.0% vs. 57.9%, *P* = 0.01). Organ donors for recipients developing PRS had a higher BMI (31.1 ± 8.2 vs. 26.4 ± 4.8, *P* < 0.001) and a higher serum sodium concentration (149.1 ± 7.8 vs. 146.1 ± 8.4, *p* = 0.04) compared to organ donors for recipients not developing PRS. Donor organs for patients who developed PRS had longer mean CITs (8.7 ± 2.3 vs. 7.8 ± 2.3 h, *p* = 0.04) and more often had an extreme CIT of ≥10 h (17 of 65 vs. 6 of 57, 26.2% vs. 10.5%, *p* = 0.02).

### Predictors of postreperfusion syndrome

In order to identify predictors of PRS we conducted a binary logistic regression analysis with PRS as dependent variable introducing known risk factors for PRS in the model (Table 2). Only visually assessed acceptable (OR 12.2 [95% CI 2.43–61.59], *P* = 0.002) or poor (OR 13.4 [95% CI 1.48–121.1], *P* = 0.02) quality of the donor organ and norepinephrine dosage before reperfusion (OR 2.2 [95% CI 1.26–3.86] per 0.1 μg kg^− 1^ min^− 1^, *P* = 0.01) predicted the occurrence of PRS. Interestingly, the histological assessment of donor organ’s steatosis was not a good predictor for PRS. This was not due to collinearity of the variables: the visual and histological assessment differed significantly (*p* < 0.001) and agreed poorly (cohen’s kappa 0.31, Table 3). For example, 21 of 122 donor organs were staged as acceptable or poor by visual inspection but classified as stage 1 steatosis by histological assessment.

**Table 2:** Predictors of PRS

	univariable				multivariable			
	Wald	OR	95% CI	*P*	Wald	OR	95% CI	*P*
Donor related								
Age [years]	1.79	1.02	0.99–1.04	0.18				
Sodium [mmol/L]	3.92	1.05	1.00–1.10	0.048	3.74	1.06	1.0–1.13	0.053
ALT [U/L]	0.94	1.00	0.99–1.00	0.76				
AST [U/L]	0.17	1.00	0.99–1.00	0.68				
Bilirubine [mg/dL]	0.28	0.92	0.68–1.24	0.60				
CIT [h]	4.09	1.19	1.05–1.41	0.04	1.62	1.14	0.93–1.38	0.20
WIT [min]	3.54	1.05	0.99–1.11	0.06				
visual assessment by surgeon^a^								
acceptable	11.03	13.14	2.87–60.03	0.001	9.21	12.23	2.43–61.59	0.002
poor	6.27	14.6	1.79–118.9	0.01	5.34	13.40	1.48–121.1	0.02
steatosis (histological)^b^								
stage 2	0.36	1.58	0.36–6.93	0.55				
stage 3	1.38	3.79	0.41–34.97	0.42				
Recepient related								
labMELD	0.05	1.00	0.97–1.04	0.82				
PoHT	6.78	2.91	1.30–6.50	< 0.01	2.15	2.07	0.78–5.48	0.14
NE dosage before reperfusion [0.1 μg kg^−1^ min^−1^]	7.86	2.00	1.23–3.24	0.01	7.56	2.20	1.26–3.86	0.01

*ALT* alanine transferase, *AST* aspartate transferase, *CIT* cold ischemia time, *WIT* warm ischemia time, *labMELD* laboratory Model of end-stage liver disease score, *PoHT* portal hypertension, *NE* norepinephrine, *OR* odds ratio, *CI* confidence interval

^a^compared to visual assessment category “good”; ^b^compared to stage 1

Results of univariable (left) and multivariable (right) binary logistic regression analyses in 122 cases of LT to predict PRS. Only visually assessed steatosis by the surgeon prior to implantation and NE dosage before reperfusion remained significant predictors of PRS in the multivariable model

**Table 3:** Differences in assessment of the graft organ between transplanting surgeon and histological examination in 122 cases of LTs

Transplanting surgeons’ macroscopic assessment	Histological assessment of the macrovesical fat content^a^			Total
	Grade 1	Grade 2	Grade 3	
Good	88	2	1	91
Acceptable	17	3	0	20
Poor	4	3	4	11
Total	109	8	5	122

^a^Grade 1: fat content 0–29%, grade 2: fat content 30–59%, grade 3: fat content ≥60%

Agreement between these two methods of assessment was poor (cohen’s kappa 0.31) and transplanting surgeons classified 21 organs as only acceptable or poor that were classified as grade 1 steatosis by histological assessment

### Postreperfusion syndrome and the intraoperative course after reperfusion

**Fig. 2:**
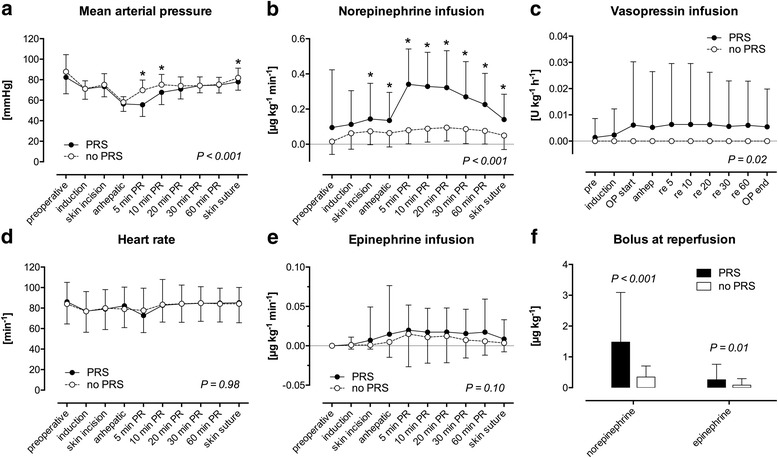
Hemodynamics. Intraoperative hemodynamics of 122 liver transplant (LT) recipients, 65 with postreperfusion syndrome (PRS, black circles), 57 without postoperative PRS (white circles). LT recipients with PRS were hemodynamically more unstable compared to patients without PRS. Mean arterial pressure after reperfusion was lower (**a**), accordingly, norepinephrine (**b**) and vasopressin infusion (**c**) were higher in LT recipients with PRS compared to recipients without PRS. Heart rate (**d**) and epinephrine infusion (**e**) did not differ. At the time of reperfusion, LT recipients with PRS received greater boli of norepinephrine and epinephrine (**f**). *P*-values: repeated measures ANOVA; *: post-hoc *t*-test; mean and standard deviation

LT recipients with PRS were hemodynamically more unstable after reperfusion until the end of surgery compared to patients without PRS (Fig. 2): Recipients with PRS had lower MAPs after reperfusion (*P* < 0.001; Fig. 2a) and accordingly received higher infusion of continuous norepinephrine (*P* < 0.001, Fig. 2b) and vasopressin (*P* < 0.001; Fig. 2c) compared to patients without PRS. Heart rate and continuous epinephrine infusion after reperfusion did not differ in LT recipients with and without PRS (Fig. 2d and e). At the time of reperfusion of the liver transplant, recipients with PRS received higher boli of norepinephrine (1.5 ± 1.6 vs. 0.3 ± 0.4 μg kg^− 1^, *P* = < 0.001) and epinephrine (0.3 ± 0.5 vs. 0.1 ± 0.2 μg kg^− 1^; *P* = 0.01; Fig. 2f).

Furthermore, LT recipients with PRS received more extensive hemotherapy intraoperatively: Compared to patients without PRS, LT recipients with PRS received more platelet concentrates (1.6 ± 1.8 vs. 1.1 ± 1.5 units; *P* = 0.04; more fibrinogen (3.0 ± 3.2 vs. 1.7 ± 1.2 g; *P* = 0.01) and more PCC (1313 ± 1610 vs. 579 ± 1133 IU; *P* = 0.01; Table 4). There was no significant difference in the amount of RBC and FFP administered as well as in other intraoperative postreperfusion adverse events (Table 4).

**Table 4:** Intraoperative adverse events

	PRS (*n* = 65)	no PRS (*n* = 57)	*P*
Hyperfibrinolysis^a^ [n]	2 (3.1%)	2 (3.5%)	1.00
Asystole [n]	0 (0.0%)	1 (1.8%)	n/a^c^
Cerebral edema^b^ [n]	0 (0.0%)	1 (1.8%)	n/a^c^
Transfusions			
RBC [U]	10.7 ± 7.3	9.5 ± 9.0	0.12
FFP [U]	18.0 ± 9.2	17.2 ± 10.3	0.50
Platelet concentrate [U]	1.6 ± 1.8	1.1 ± 1.5	0.04
Fibrinogen [g]	3.0 ± 3.2	1.7 ± 2.6	0.01
PCC [IU]	1313 ± 1610	579 ± 1133	0.01

*PRS* postreperfusion syndrome, *RBC* red blood cell concentrate, *FFP* fresh frozen plasma, *PCC* prothrombin complex concentrate

^a^detected by thrombelastometry; ^b^detected by mydriasis after reperfusion; ^c^too little events to calculate *P*; intraoperative adverse events after reperfusion in 122 liver transplant recipients apportioned by patients with and without postreperfusion syndrome (PRS)

### Postreperfusion syndrome and the postoperative course after transplantation

**Fig. 3:**
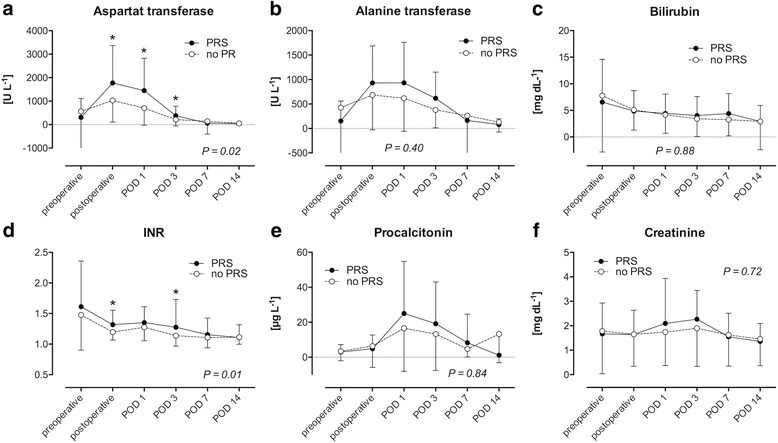
Clinical chemistry. Postoperative clinical chemistry of 122 liver transplant (LT) recipients, 65 with postreperfusion syndrome (PRS, black circles), 57 without postoperative PRS (white circles). LT recipients with PRS suffered from greater postoperative transplant damage and poorer transplant function during the postoperative course: Aspartat transferase blood concentration was higher (**a**) and blood hemostasis was poorer (**d**) compared to LT recipients without postreperfusion syndrome (alanine transferase was higher but did not reach significance, (**b**) LT recipients with and without PRS did not differ in gall retention (**c**). Inflammation marker procalcitonin (**e**) and kidney function marker creatinine (**f**) were peaking higher during the postoperative course in LT recipients with PRS compared to recipients without PRS but did not reach statistical significance. P-values: repeated measures ANOVA; *: post-hoc *t*-test; mean and standard deviation

LT recipients with PRS demonstrated a more severe organ damage after transplantation: During the first three postoperative days, AST was higher in patients with PRS (*P* = 0.02; Fig. 3a), as was ALT, but without statistical significance (*P* = 0.40; Fig. 3b). LT recipients with and without PRS did not differ in bile retention, as postoperative serum bilirubin concentrations were similar (Fig. 3c). Recipients with PRS postoperatively had poorer organ function as hemostasis was poorer compared to recipients without PRS (*P* = 0.01; Fig. 3d). The inflammation marker procalcitonin as well as the kidney function marker serum creatinine did not differ in LT recipients with and without PRS (Fig. 3e and f).

**Fig. 4:**
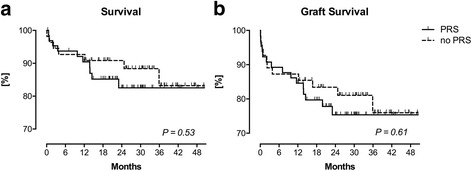
Survival. Kaplan-Meier survival analysis of 122 liver transplant (LT) recipients, 65 with postreperfusion syndrome (PRS; solid line) and 57 with PRS (broken line). Survival analyses were conducted for LT recipient survival (**a**) as well as graft survival (retransplantation or recipient death; (**b**) PRS: postreperfusion syndrome

Regarding postoperative adverse events, LT recipients with PRS suffered more often from postoperative delirium (8/65 vs. 1/57, 12.3% vs. 1.8%; *P* = 0.04) and major cardiovascular events (11/65 vs. 3/57, 16.9% vs. 5.3%; *P* = 0.05; Table 5). LT recipients with PRS also tended to have a longer ICU LOS (11.7 ± 17.0 vs. 9.7 ± 15.7; *P* = 0.09; Table 5). A Kaplan-Meier survival analysis for LT recipient survival (Fig. 4a) and graft survival (Fig. 4b) did not show significant differences.

**Table 5:** Postoperative outcome

	PRS (*n* = 65)	no PRS (*n* = 57)	*P*
Early allograft dysfunction [n]	25 (38.5%)	16 (28.1%)	0.25
Retransplantation [n]	5 (7.7%)	4 (7.0%)	0.75
Due to primary non function^a^ [n]	3 (4.6%)	1 (1.8%)	
Due to thrombosis [n]	0 (0.0%)	1 (1.8%)	
Acute rejection [n]	12 (18.5%)	13 (22.8%)	0.66
Surgical revision [n]	26 (40.0%)	25 (43.9%)	0.72
Bleeding [n]	22 (33.8%)	19 (33.3%)	1.00
Severe infection/sepsis [n]	11 (16.9%)	8 (14.0%)	0.80
Renal function [n]			
RRT [n]	27 (41.5%)	20 (35.1%)	0.27
Major cardiovascular events^b^ [n]	11 (16.9%)	3 (5.3%)	0.05
Adverse CNS events [n]			
Delirium [n]	8 (12.3%)	1 (1.8%)	0.04
Intracranial bleeding [n]	3 (4.6%)	0 (0.0%)	0.25
Seizures [n]	1 (1.5%)	1 (1.8%)	1.00
Extubation in the OR [n]	15 (23.1%)	20 (35.1%)	0.16
ICU LOS [days]	11.7 ± 17.0	9.7 ± 15.7	0.09
Hospital LOS [days]	36.5 ± 18.2	35.6 ± 23.9	0.24
Deaths [n]	10 (15.4%)	7 (12.3%)	0.33
Due to sepsis/MOF [n]	6 (9.2%)	6 (10.5%)	
Due to cardiovascular events [n]	2 (3.1%)	1 (1.8%)	
Due to bleeding [n]	1 (1.5%)	0 (0.0%)	
Due to carcinoma [n]	1 (1.5%)	0 (0.0%)	

Short-term postoperative outcome of 122 liver transplant recipients with and without postreperfusion syndrome (PRS); *RRT* renal replacement therapy, *CNS* central nervous system, *ICU* intensive care unit; LOS: length of stay *MOF* multi organ failure *PRS* postreperfusion syndrome

^a^Primary non-function: re-transplantation or death within 7 days

^b^major cardiovascular events: asystole, resuscitation, non ST elevation myocardial infarction

## Discussion

In this single center retrospective analysis of 122 LT recipients, we found that approximately half of the patients suffered from PRS (53.3%). Multivariable regression analysis identified two predictors of PRS: Only visually assessed acceptable (OR 12.2, *P* = 0.002) and poor (OR 13.4, *P* = 0.02) donor organ quality by the transplanting surgeon and intraoperative norepinephrine infusion rate before reperfusion of the liver transplant (OR 2.2 per 0.1 μg/kg/min, *p* = 0.01) were associated with the occurrence of PRS. LT recipients suffering from PRS were hemodynamically more instable after reperfusion compared to recipients not suffering from PRS. They had lower MAPs from reperfusion until the end of surgery (*P* < 0.001). Hence, they received more epinephrine and norepinephrine before reperfusion (*P* < 0.001 and *P* = 0.01, respectively) and higher rates of continuous infusion of norepinephrine (*P* < 0.001) and vasopressin (*P* = 0.02) after reperfusion of the transplant. Moreover, LT recipients with intraoperative PRS had a more complicated postoperative course compared to recipients without intraoperative PRS: They had more adverse cardiac events (*P* = 0.05) and suffered more often from postoperative delirium (*P* = 0.04).

All 122 LT recipients were operated at the same center with the same technique (venovenous/portalvenous bypass) and with uniform anesthesiologic management, guided by a SOP that included instructions for the management of PRS. No changes in operating technique or anesthesiologic management were made during the study period, leading to a homogenous single center study sample. Nevertheless, this study has several weaknesses. Due to the retrospective design of the analyses, data quality could be impaired. Histological data for our analyses was limited to steatosis assessment. Analyses of hemodynamics from hand-written anesthesia protocols are often suspected to present “sugar-coated” hemodynamic values. Although we cannot rule out that this was the case, our data present clinically and empirically plausible hemodynamics. At worst, hemodynamic stability is overestimated and the effects shown are even more pronounced. The fact that this was a single center analysis combined with the uncommon but consistent use of intraoperative venovenous/portalvenous bypass limits the external validity of our results.

Although rather on the top end of the range, PRS incidence of 53% in our sample concurs with several other studies [8, 10, 12, 17, 19]. The pathophysiology of PRS is complex and not entirely understood. The abrupt influx of cold, hyperkalemic and acidic blood into the circulation, air or thrombotic embolization and the release of vasoactive substances from the graft liver contribute to PRS [29–31]. The rationale for using a venovenous/portalvenous bypass during the anhepatic phase at our center is to ensure maximum safety of the procedure [24]. The combination of a femoro-brachial and porto-axillary bypass reduces lower limb and mesenterial congestion and therefore reduces the abrupt influx of hyperkalemic and acidic blood into systemic circulation at the time of reperfusion. Nevertheless, the use of a bypass during LT surgery remains controversial [13, 32, 33]. The PRS effect seen in our study is therefore likely to be predominantly caused by vasoactive substances released from the graft. With that in mind, PRS incidence does appear rather high.

A possible explanation for this contradiction may be the fact that we used an extended definition for PRS. PRS is commonly defined as a decrease in MAP of more than 30% from the baseline value for more than one minute during the first five minutes after reperfusion [7, 8, 10–12]. We extended the definition for PRS for mainly two reasons. First, the treatment of PRS seeks to preserve hemodynamic stability. At the time of reperfusion, anesthesiologists expect a certain degree of hemodynamic instability and preemptively treat a (soon to be) falling MAP with catecholamines [11], either as bolus or by increasing continuous infusion of the very same. Since this preemptive treatment conceals the occurrence of by-definition-PRS, we added (i) the administration of an intravenous bolus of norepinephrine > 2 μg kg (BW) ^-1^ as well as (ii) the increase of continuous norepinephrine infusion of ≥0.1 μg kg BW^− 1^ within the first 5 min after reperfusion as new criteria to the definition. Second, our clinical empiricism and the literature have presented cases of prolonged vasodilation after reperfusion [34–36]. We thus included (iii) an increase of continuous norepinephrine infusion up to 30 min after reperfusion and (iv) the initiation of continuous vasopressin infusion after reperfusion as additional criteria for PRS. This altered definition impedes comparability with other studies and may overestimate PRS incidence compared to the commonly used definition. However, our data appear clinically plausible and concur with description of hemodynamic recovery after PRS by other authors [17].

In this study, multivariable regression modelling identified one donor related and one recipient related predictor of PRS: graft quality – visually assessed by the transplanting surgeon – and cumulative intraoperative norepinephrine dosage before reperfusion of the liver graft. The variety of risk factors and risk prediction models described in different studies suggests that PRS may occur in an unpredictable manner or may be highly subjective to center-specific effects [8, 13–20, 22]. The most frequent reported risk factors for PRS are longer CIT [15, 16, 18, 20] and intraoperative hemodynamics prior to reperfusion [14, 15, 17, 20], followed by graft steatosis [8, 16], operation time and technique [15, 18], severity of recipient’s liver disease [8, 19], and donor age [13, 17]. One single center study retrospective analysis was unable to identify even a single risk factor in a retrospective analysis of a sample of 261 LT recipients [37]. Our findings that graft quality and hemodymic impairment prior to reperfusion are risk factors for PRS concur with several similar studies. However, it remains cryptic why the multitude of these observational studies generate such a diversity of findings when it comes to predicting postreperfusion syndrome. Prospective multi-center trials, the creation of an LT register or meta-analyses investigating the prediction of PRS could help to shed light on this matter in the future.

The increasing number of liver transplantations has led to an increased demand of donor organs [1] and to the growing use of organs from extended criteria donors (ECD) [23]. A common quality marker for organs is fatty change, or steatosis. Steatosis has indeed been linked to the occurrence of PRS [8, 16]. Studies have shown that transplantation of liver grafts with moderate to severe steatosis can very well be conducted without sacrificing recipient survival but in particular, that increased efforts are needed when dealing with these organs [26, 38]. However, macroscopic assessment of the organ may not be qualified to assess steatosis: Rey and colleagues have examined 36 livers of organ donors which were explanted but not allocated. The authors found that macroscopic appearance and coloring compared to histopathologic evaluation for steatosis unveiled discrepancies: Most of the graft livers with histopathologically confirmed mild degree of steatosis macroscopically appeared yellow, thus of false poor quality and could have been transplanted. In our analyses, microscopic steatosis evaluation was a worse predictor for PRS than the visual evaluation of the transplanting surgeon. This may be the case because surgeons likely assess the organ not only by its color, but also by general appearance, organ consistency, and size. It may even be the case that information of the recipient’s medical history and the knowledge that the organ was already rejected once by another center or had a long CIT additionally influenced the assessment. Therefore, the multitude of information may have enabled the surgeon to predict PRS better than any single variable (recipient status, CIT, steatosis, etc.).

We also found intraoperative norepinephrine infusion rate *before* reperfusion to be a predictor of PRS. This also concurs with other studies that have identified markers of hemodynamic instability prior to reperfusion as predictors of PRS. One possible explanation for norepinephrine infusion or hemodynamic instability is hypovolemia. This would also concur with other studies that have shown lower central venous pressure at time of dissection [14] or at time of reperfusion [17] as well as a higher requirement for transfusion units [15] to be predictors of PRS. A second possible explanation is vasodilatation or vasplegia which has been linked to end stage liver disease [39, 40]. Case reports have described vasoplegic syndrome to occur during liver transplantation [31, 41]. However, differentiation between the two can sometimes be complicated as both appear alike [34]. A third possible explanation may be that these patients already had a compromised cardiac function. E.g., Xu and colleagues could link left ventricular diastolic dysfunction to the occurrence of PRS in a cohort of 330 Chinese LT recipients [20]. Unfortunately, our data did not contain systematic and detailed cardiac function assessment of the recipients and the literature lacks systematic investigation of its impact on the occurrence of PRS. Moreover, it has been suggested by several authors that PRS could be only a sign of an underlying problem. It could be that anhepatic hypovolemia, vasoplegia and/or impaired cardiac function demask as PRS intraoperatively. On top of it, patients receiving a LT from ECD appear especially susceptible to severe PRS. In this light, these patients should especially be optimized regarding hemodynamics and acid-base balance when awaiting reperfusion.

Similar to the prediction of PRS, data on the effect of PRS on LT recipients’ outcome is inconclusive. In particular, the impact of PRS on hard clinical endpoints such as primary graft nonfunction with requirement for retransplantation and mortality is unresolved. E.g., some studies show an effect of PRS on mortality [15, 18–20] and others did not [13, 17]. In fact, we were not able to show a significant association of PRS with primary non-function (4.6 vs. 1.8%) and mortality (15.4% vs. 12.3%) in this study population. This is most likely due to the small sample size, again underscoring the need for multicenter registries. Regarding post-transplantation morbidity, we could demonstrate in our sample that the occurrence of PRS was associated with a higher frequency of postoperative delirium (12.3% vs. 1.8%) and major cardiovascular events (16.9% vs. 5.3%). While other studies have demonstrated early allograft dysfunction [42, 43], ICU length of stay [43] and the need for postoperative renal dysfunction [19, 20] we could not substantiate these associations in our study sample.

## Conclusions

The data of this study demonstrated that both the visual assessment of the liver graft by the transplanting surgeon as well as the intraoperative recipients need for catecholamines before reperfusion of the graft were predictors for PRS at our center. Besides the known risk factors for PRS, anesthesiologists should keep these newly described risk factors in mind when preparing for reperfusion. As the impact of PRS on short- and long-term hard clinical endpoint remains inconclusive, further investigations and possibly multicenter prospective registries could ultimately resolve the impact of PRS on LT recipients’ outcome.

## Abbreviations

ALT: Alanine transferase
AST: Aspartat transferase
CIT: Cold ischemia time
ECD: Extended criteria donors
FFP: Fresh frozen plasma
INR: International normalized ratio
labMELD: Laboratory Model of end stage liver disease
LT: Liver transplantation
MAP: Mean arterial pressure
MELD: Model of end stage liver disease
min: Minute(s)
NE: Norepinephrine
OR: Odds ratio
PC: Platelet concentrate
PCC: Prothrombin complex concentrate
POD: Postoperative day
PoHT: Portal hypertension
PRS: Postreperfusion syndrome
PSC: Primary biliary cirrhosis
RBC: Red blood cell concentrate
SOP: Standard operating procedure
TEM: Thrombelastometry
US: United States
WIT: Warm ischemia time
